# Supplementary material for: Intimate Partner Violence (IPV) in Military and Veteran Populations: A Systematic Review of Population-Based Surveys and Population Screening Studies
Source: Int J Environ Res Public Health. 2022 Jul 21;19(14):8853. doi: 10.3390/ijerph19148853 (PMC9316917; doi:10.3390/ijerph19148853)
Supplement: Supplementary file 1 [file ijerph-19-08853-s001.zip › ijerph-1776469-supplementary.pdf]

Table S1. Example Search Strategy Conducted in PsycINFO Database**.**

Step	Search Terms	No of Records
S1	battered females/ or domestic violence/ or intimate partner violence/	21,513
S2	(IPV OR "intimate partner violence" OR "partner violence" OR "spousal violence" OR "domestic violence" OR "intimate partner abuse" OR "partner abuse" OR "spouse abuse" OR "intimate partner victim*" OR "intimate partner perpetrat*" OR "family violence")ab,mh,ti.	22,811
S3	((Abus* OR batter* OR violen* OR beat* or assault*) adj3 (perpetrat* OR victim* OR domestic OR partner* OR famil* OR spous* OR wom*n OR m*n OR wife OR wives OR husband* OR gender)).ab,ti.	48,376
S4	S1 or S2 or S3	51,509
S5	military personnel/ or air force personnel/ or army personnel/ or coast guard personnel/ or marine personnel/ or military veterans/ or national guard personnel/ or navy personnel/	28,344
S6	("ex-military" OR veteran* OR military OR "active duty" OR "military personnel" OR army OR navy OR marines OR "air force" OR "armed forces" OR "military service personnel" OR "soldier").ab,mh,ti.	52,459
S7	S5 or S6	55,355
S8	epidemiology/ or population/	53,347
S9	prevalence.mp.	125,613
S10	(prevalence OR prevalent OR occurrence OR incidence OR epidemiol* OR population OR representative).ab,mh,ti.	506,520
S11	(random* NEAR/3 (select* or sampl*)).ab,ti.	36,502
S12	S8 or S9 or S10 or S11	538,246
S13	Exp mental health services/	41,545
S14	treatment/ or intervention/ or prevention/	159,289
S15	("Health*care setting*" or "Health*care service*" or "Health*care system*" or "Health*care centre*" or "Health setting*" or "Health service**" or "Health system" or "Health centre*" or "clinical setting" or "clinical service").ab,mh,ti.	89,725
S16	(Implement* or Treat* or program* or Interven* or Prevent* or Assess* or Screen* or "Veterans Health Administration" or VHA or Patient or provider or Clinic).ab,mh,ti.	1,978,471
S17	S13 or S14 or S15 or S16	2,016,433
S18	S12 or S17	2,234,432
S19	S4 and S7 and S18	799
S20	limited S19 to (peer reviewed journal and English language)	554

Notes. ab = abstract, mh = mesh headings, ti = title.

Table S2. JBI Critical Appraisal Checklist for Studies Reporting Prevalence Data**.**

Item	Question	Criteria
1	Was the sample frame appropriate to address the target population?	To receive a score of 1 ("Yes"), the authors must use a sample frame that targets active duty personnel or veterans.
2	Were study participants sampled in an appropriate way?	To receive a score of 1 ("Yes"), the authors must utilize probability-based sampling strategies (i.e., random, stratified or systematic sampling) or identification strategies involving systematic screening of target populations.
3	Was the sample size adequate?	To receive a score of 1 ("Yes"), the authors must provide a sample size calculation. Large national surveys without power calculations also received a score of 1. If no sample size calculation was provided and the study did not use a large national survey, a rating of 0 ("Unclear") was given.
4	Were the study subjects and the setting described in detail?	To receive a score of 1 ("Yes"), the authors must describe the sample in sufficient detail (e.g., gender, age and other sociodemographic variables).
5	Was the data analysis conducted with sufficient coverage of the identified sample?	To receive a score of 1 ("Yes"), the authors must account for coverage bias (e.g., by utilizing random, stratified, or systematic sampling). Studies that only included married individuals were given a score of 0 ("No").
6	Were valid methods used for the identification of the condition?	To receive a score of 1 ("Yes"), the authors must use validated measures of IPV. Non-validated and single item measures received a score of 0 ("No").
7	Was the condition measured in a standard, reliable way for all participants?	To receive a score of 1 ("Yes"), the authors must use reliable measures of IPV. Single item measures received a score of 0 ("No").
8	Was there appropriate statistical analysis?	To receive a score of 1 ("Yes"), the authors must report the percentage, the confidence intervals around the percentage, as well as the numerator and denominator used to calculate the percentage.
9	Was the response rate adequate, and if not, was the low response rate managed appropriately?	To receive a score of 1 ("Yes"), the response rate must be greater than 60%. If the response rate was less than 60%, authors could receive a score of 1 if they used statistical weights in the analyses to account for the low response.

Notes. For more details regarding each item see [34].

Table S3. Summary of eligible studies (*n* = 31 [a]).

	Perpetration	Victimisation
***N* studies (n participants)**	12 (131,777)	25 (140,689)
**Gender**		
Male only	4	3
Female only	1	12
Combined	7	10
**Country**		
US	10	21
Other	2	4
**Study design**		
Cross-sectional	9	22
Longitudinal	2	1
Routine screening	1	2
**Sampling strategy**		
Probability	9	23
Population-screening	3	2
**Setting**		
General military/community-based	9	16
Military health service	3	9
**Serving Status**		
Veteran	7	8
Active duty personnel	5	16
Combined	0	1
**Era**		
Pre-2001	2	2
Post-2001	8	8
Mix era	2	2
Not reported	0	13
**Timepoint**[b]		
Recent IPV	11	14
Lifetime IPV	3	11
**Types of IPV**		
Any IPV	12	25
Physical	10	17
Severe physical	2	1
Sexual	2	11
Psychological	2	14
Physical and/or sexual	1	3
Other	2	6
**Assessment of IPV**		
Validated tool	8	9
Validated screening tool	0	6
Non-validated tool	4	11

Notes. [a] Findings from two unique samples were reported in a single paper [40] were counted as separate studies. [b] Recent IPV includes studies that reported IPV prevalence within the past year, past 6-months, past month, or within the current relationship. Lifetime IPV includes all other timepoints. Two studies reported estimates of both recent and lifetime IPV perpetration.

Table S4. Characteristics of studies of IPV impact and context (including coercive and controlling behaviours).

Study	Country	Sample	Design	IPV Dimension	Measure	Key Findings
Bartlett [55]	U.S.	*n* = 642 veterans, who were all men	Population-based survey (online). Male veterans who reported trauma exposure randomly selected from a larger study involving an online panel which was representative of U.S. population.	Fear of partner victimisation.	Fear of partner item from the HARK screening instrument.	5.4% of male veterans reported fear of partner in the past year.
Creech [45]	U.S.	*n* = 102 veterans (51% army), who were all women.	Population-based survey (postal). Women veterans who were in intimate relationships sampled randomly from a VHA database of veterans involved in Iraq and Afghanistan.	Impact of IPV perpetration.<br><br>Impact of IPV victimisation.	Injury subscale of the CTS-2 perpetration version.<br><br>Injury subscale of the CTS-2 victimisation version.	(1) Around 2.0% of participants reported having caused injury to their partner.<br><br>(2) Around 2.0% of participants reported their partner had caused injury.
Dighton [62]	UK	*N* = 257 veterans (81.7% male)	Population-based survey (interview). Analyses of data from participants who self-identified as veterans in an epidemiological survey.	Partner control of money victimisation.	Single item regarding whether the participant had ever had money withheld by their partner.	There were 7.9% of men and 18.7% of women that reported that they had ever had money withheld by their partner.
Foran [46]	U.S.	*n* = 42,744 AD personnel (100% air force)	Population-based survey (online) conducted in 2006. Stratified sample of AD personnel across air force installations worldwide. Analyses were limited to participants who were currently in an intimate relationship.	Clinically significant physical IPV perpetration and victimisation.<br><br>Clinically significant emotional abuse victimisation.	15 item pairs (perpetration and victimisation) similar to the Physical Assault subscale of the CTS-2, with 8 follow-up questions about resulting injuries. Clinically significant IPV defined by acts of physical violence associated with high inherent dangerousness (e.g., use of a weapon), or physical injury.<br>A preliminary question asked about significant depression, stress, and/or fear resulting from their partners behaviour, with affirmative responses followed by 10 items about	(1) 4.7% of men and 3.3% of women reported perpetration of physical IPV with impact in the past year.<br><br>(2) 3.5% of both men and women reported exposure to physical IPV with impact in the past year.<br><br>(3) 6.0% of men and 8.5% of women reported exposure to clinically significant emotional abuse in the past year.

Study	Country	Sample	Design	IPV Dimension	Measure	Key Findings
					emotionally abusive behaviours. If any acts were reported then additional items also asked whether these had contributed to depression / stress / fear. Clinically significant emotional abuse victimisation defined by exposure to at least one act that resulted in fear, stress, or depression that interfered with functioning.	
Hundt [44]	U.S.	*n* = 264 mixed-era veterans, who were 91% male	Analyses of health services data from routine assessments conducted during referral to a VHA mental health clinical.	Veteran-reports of their partners' fear of anger	Single item: "Has you partner ever been afraid of your anger in the past year".	42% of veterans reported that their partner had been afraid of their anger in the past year.
Iverson [63]	U.S.	*n* = 411 veterans, who were all women.	Population-based survey (online). Women veterans recruited from a probability-based survey panel that was representative of most U.S. households.	Intimate partner stalking victimisation.	Single item regarding lifetime exposure to harassing behaviours by a partner or ex-partner that caused fear or safety concerns on two or more occasions.	64.4% of women veterans that reported lifetime exposure to intimate partner stalking.
Kimerling [65]	U.S.	*n* = 6,287 veterans, who were all women.	Population-based survey (telephone). Women veterans sampled from a database of records of women veterans who had visited VHA primary care services in last fiscal year.	Fear of partner victimisation	Fear of partner item from the HARK screening instrument.	Rates of past year fear of partner were 14.4% (aged 18-30), 12.8% (aged 31-44), 11.7% (aged 45-54), 8.1% (aged 55-64), and 2.1% (aged 65+ years).
Lorber [40]	U.S.	*n* = 25,285 AD personnel (100% air force)	Population-based survey (online) conducted in 2008. Stratified random sample of AD personnel across air force installations worldwide. Analyses limited to participants who were currently in intimate relationships, and had one or more children living in the household.	Clinically significant physical IPV perpetration and victimisation.	Partner physical abuse perpetration and victimisation moduled of Family Maltreatment Measure (FM). This comprises 15 item pairs (perpetration and victimisation) similar to Physical Assault subscale of the CTS-2, with follow-up questions about injuries. Clinically significant physical IPV operationalised by physical violence	(1) 1.1% of AD personnel reported clinically significant physical IPV perpetration in the past year.<br><br>(2) 2.0% of AD personnel reported clinically significant physical IPV victimisation in the past year.

Study	Country	Sample	Design	IPV Dimension	Measure	Key Findings
					associated with injury, or high inherent dangerousness (e.g., use of a weapon).	
				Clinically significant emotional abuse victimisation.	Partner psychological IPV victimisation module of the FM. This comprises preliminary questions about significant depression, stress, and/or fear resulting from their partners behaviour, with affirmative responses followed by 10 items about past year frequency of psychologically abusive behaviours. Clinically significant emotional abuse operationalised by reports of abusive behaviour associated with fear, stress, or depression that interfered with functioning.	(3) 7.2% of AD personnel reported clinically significant emotional abuse victimisation in the past year.
Lorber [40]	U.S.	n = 29,359 AD personnel (100% air force)	Population-based survey (online) conduced in 2011. Stratified random sample of AD personnel across air force installations worldwide. Analyses limited to participants who were currently in intimate relationships, and had children living in the household.	Clinically significant physical IPV perpetration and victimisation.	Partner physical abuse perpetration and victimisation module of the FM.	(1) 0.5% reported clinically significant physical IPV perpetration in the past year.<br>(2) 1.4% reported clinically significant physical IPV victimisation in the past year.
				Clinically significant emotional abuse victimisation.	Partner psychological IPV victimisation module of the FM.	(3) 7.0% reported clinically significant emotional abuse victimisation in past year.
Rosenfeld [67]	U.S.	n = 1,241 women veterans who had	Population-based survey (interview). Random sample of women	Reproductive coercion (victimisation).	Survey items asking if male partners in had taken off condom during sex or refused to use a condom (so their	11.0% of women reported reproductive coercion in the past year.

Study	Country	Sample	Design	IPV Dimension	Measure	Key Findings
		sex with a man in past year.	veterans who had recently used VHA primary care services.		partner would get pregnant), or withheld or restricted use of birth control.	
Zamorski [47]	Canada	n = 1,745 AD personnel, who were in an intimate relationship.	Population-based survey (postal). Secondary analyses of a general health survey of a random sample of AD personnel identified through the Canadian Armed Forces human resources system.	(1) Limits contact with friends / family (victimisation) (2) Demanded to know 'who and where' at all times (perpetration and victimisation). (3) Damaged or destroyed possessions / property (victimisation).	Survey items addressing specific abusive behaviours based on the Canadian General Social Survey on Victimisation. The reference period for items was over the life of the current relationship.	(1) 6.0% of men reported their partner limited contact with friends and family (rates were low among women and not reported) (2) 8.2% of men (5.7% of women) reported their partner demanded to know 'who and where' they were at all times, while 3.8% of men reported parallel figures for perpetration (not reported for women). (4) 4.6% of men (4.1% of women) reported their partner had damaged or destroyed possessions or property.

Notes. AD = Active duty; CTS-2 = Conflict Tactic Scale – 2; HARK = Humiliation, Afraid, Rape, Kick; FM = Family Maltreatment Measure; VHA = Veteran Health Administration.

Table S5. Risk of bias of eligible studies**.**

Author	Q1	Q2	Q3	Q4	Q5	Q6	Q7	Q8	Q9	Overall
Albright [53]	Yes	Unclear	Yes	Yes	Unclear	No	No	No	Unclear	3/9
Albright [54]	Yes	Yes	Yes	Yes	Yes	No	No	Yes	Yes	7/9
Bartlett [55]	Yes	Yes	Yes	Yes	Yes	Yes	Yes	No	Yes	8/9
Belik [56]	Yes	Yes	Yes	No	Yes	No	No	Yes	Yes	6/9
Bostock [42]	Yes	Yes	Unclear	Yes	Yes	Unclear	Unclear	No	No	4/9
Campbell [57]	Yes	Yes	Unclear	Yes	Yes	Yes	Yes	No	No	6/9
Campbell [58]	Yes	Yes	Unclear	Yes	Yes	Yes	Yes	No	Yes	7/9
Cancio [49]	Yes	Unclear	Yes	Yes	Unclear	Unclear	Unclear	No	Unclear	3/9
Cerulli [59]	Yes	Yes	Yes	Yes	Yes	No	No	No	Yes	6/9
Creech [45]	Yes	Yes	Unclear	Yes	No	Yes	Yes	No	No	5/9

Author	Q1	Q2	Q3	Q4	Q5	Q6	Q7	Q8	Q9	Overall
Dichter [60]	Yes	Yes	Yes	Yes	Yes	No	No	No	Unclear	5/9
Dichter [6]	Yes	Yes	Unclear	No	Yes	Yes	Yes	No	Unclear	5/9
Dichter [61]	Yes	Yes	Yes	Yes	Yes	Yes	Yes	No	Unclear	7/9
Dighton [62]	Yes	Yes	Unclear	Yes	Yes	No	No	No	Yes	5/9
Foran [46]	Yes	Yes	Yes	Yes	Yes	Yes	Yes	No	Yes	8/9
Gerlock [48]	Yes	Yes	Unclear	Yes	Yes	No	No	No	Unclear	4/9
Hundt [44]	Yes	Yes	Unclear	Yes	Yes	No	No	No	Unclear	4/9
Iverson [5]	Yes	Yes	Unclear	Yes	Yes	Yes	Yes	No	Yes	7/9
Iverson [63]	Yes	Yes	Yes	Yes	Yes	Yes	Yes	No	Yes	8/9
Iverson [64]	Yes	Yes	Unclear	Yes	Yes	Yes	Yes	No	Yes	6/9
Kimerling [65]	Yes	Yes	Yes	Yes	Yes	Yes	Yes	No	Yes	8/9
Lorber [40]	Yes	Yes	Yes	Yes	No	Yes	Yes	No	Yes	7/9
McCarroll [50]	Yes	Unclear	Yes	Yes	No	Yes	Yes	No	Yes	6/9
McCarroll [51]	Yes	Yes	Yes	Yes	No	Yes	Yes	No	Unclear	6/9
Mercado [66]	Yes	Yes	Unclear	Yes	Yes	Unclear	Unclear	No	Yes	5/9
Ortabag [43]	Yes	Yes	Unclear	Yes	Yes	No	No	No	Yes	5/9
Rosenfeld [67]	Yes	Yes	Yes	Yes	Yes	No	No	No	No	5/9
Sadler [68]	Yes	Yes	Yes	Yes	Yes	Unclear	Unclear	No	Yes	6/9
Sadler 2004 [41]	Yes	Yes	Yes	Yes	Yes	Unclear	Unclear	No	Yes	6/9
Schmaling [52]	Yes	Yes	Yes	Yes	No	Yes	Yes	No	Yes	7/9
Skomorovsky [69]	Yes	Yes	Unclear	Yes	Yes	No	No	No	No	4/9
Zamorski [47]	Yes	Yes	No	Yes	Yes	Yes	Yes	Yes	Yes	8/9

Notes. The risk of bias rating for each criterion was categorised Yes = 1, No = 0, and Unclear = 0. Total scores generated were utilised to generate the overall risk of bias rating (i.e., score ≥ 6 = low risk; < 6 = high risk). Q1= Was the sample frame appropriate to address the target population? Q2 = Were study participants sampled in an appropriate way? Q3 = Was the sample size adequate? Q4 = Were the study subjects and the setting described in detail? Q5 = Was the data analysis conducted with sufficient coverage of the identified sample? Q6 = Were valid methods used for the identification of the condition? Q7 = Was the condition measured in a standard, reliable way for all participants? Q8 = Was there appropriate statistical analysis? Q9 = Was the response rate adequate, and if not, was the low response rate managed appropriately? For more details on each item, please see Munn et al. (2020).

Table S6. Sensitivity analysis comparing studies classified as low risk of bias and high risk of bias for any current IPV perpetration and any current IPV victimisation.

	Low Risk of Bias			High Risk of Bias		
	k	Estimate (95% CI)	I^2	k	Estimate (95% CI)	I^2
Any current IPV perpetration	7	6.2% (2.0-17.4%)	100%	4	37.0% (18.2-60.7%)	98.1%
Any current IPV victimisation	9	16.8% (9.6-27.7%)	99.9%	5	29.4% (14.9-49.7%)	99.3%

Table S7. Sensitivity analysis comparing the original analysis of any current IPV perpetration and any current IPV victimisation, and the analyses with outliers removed.

	Original analysis			Outliers removed		
	**k**	**Estimate (95% CI)**	**I^2**	**k**	**Estimate (95% CI)**	**I^2**
Any current IPV perpetration	11	12.7% (4.9-29.0%)	100%	7[a]	16.0% (12.0-21.1%)	99.5%
Any current IPV victimisation	14	20.7% (13.1-31.1%)	99.9%	7[b]	20.2% (17.3-23.5%)	96.3%

Notes. Four outliers were detected in the analysis of any current IPV perpetration, with two extremely small effects (Lorber et al., 2018) and two extremely large effects (Creech et al., 2017; Hundt & Holohan, 2012). Seven outliers were detected in the analysis of any current IPV victimisation, with four extremely small effects (Dichter, Sorrentino, et al., 2017; Lorber et al., 2018; Rosenfeld et al., 2018) and three extremely large effects (Albright et al., 2019; Creech et al., 2017; Iverson et al., 2017).
